# Supplementary material for: MetaRibo-Seq measures translation in microbiomes
Source: Nat Commun. 2020 Jun 29;11:3268. doi: 10.1038/s41467-020-17081-z (PMC7324362; doi:10.1038/s41467-020-17081-z)
Supplement: Supplementary file 10 — Supplementary Data 7 [file 41467_2020_17081_MOESM10_ESM.zip › File2/Confidence_High_Taxonomy/163391_out.krona.html]

Javascript must be enabled to view this page.

members
magnitude
magnitudeUnassigned
count
unassigned
taxon
rank

163391\_out

3

superkingdom
2
3

1239
3
phylum

186801
3
class

186802
3
order

family
3
186803

genus
3
841

3
301302

SRS020394\_contig\_number\_contig-100\_7397.47977SRS1055056\_contig\_number\_contig-100\_877.78077SRS893341\_contig\_number\_16309
species
